# Assessing antibiotics consumption, use and outcomes in a Yemeni tertiary hospital: A prospective cross-sectional study

**DOI:** 10.1371/journal.pone.0330714

**Published:** 2025-08-22

**Authors:** Adel Ahmed Alshaikh, Mohammed Abdullah Kubas

**Affiliations:** 1 Department of Clinical Pharmacy and Medical Sciences, Lebanese International University, Sana’a, Yemen; 2 Department of Clinical Pharmacy & Pharmacy Practice, University of Science and Technology, Sana’a, Yemen; The University of Lahore, PAKISTAN

## Abstract

**Background:**

Antibiotics (ABs) have saved countless lives, but their misuse has led to a serious problem: antibiotic resistance. This growing phenomenon poses serious threats to public health worldwide, as it could make treating infections significantly more difficult in the future.

**Objectives:**

This study aimed to investigate antibiotic consumption and use patterns in a tertiary hospital in Sana’a, Yemen, by comparing Prescribed Daily Doses (PDD) to Defined Daily Doses (DDD), and identifying factors associated with antibiotic misuse and its impact on patient outcomes.

**Methods:**

A prospective cross-sectional study was conducted among adult inpatients in a tertiary hospital in Sana’a, Yemen over two months (January 12 to March 11, 2024), involving 597 patients. Data on antibiotic prescriptions, patient demographics, and outcomes were collected.

**Results:**

A high prevalence of antibiotic use was observed (92.5%), with a notable proportion of prescriptions from the “Watch” category (56.7%). Significant PDD-DDD deviations were common, encompassing both overuse (36.8%) and underuse (63.2%). Factors associated with antibiotic deviations included patient age (26–44 years), gender (female), and ward type (private). The most commonly prescribed antibiotics were Ceftriaxone (33.6%), Metronidazole (21.8%), Vancomycin (6.0%), Levofloxacin (4.9%), Imipenem/Cilastatin (4.7%), and Moxifloxacin (3.6%). Notable deviations from DDD were observed for Levofloxacin (overuse by 28%), Imipenem/Cilastatin (underuse by 40.5%), and other agents. Antibiotic misuse was associated with longer hospital stays and less favorable discharge outcomes.

**Conclusion:**

The study found an alarmingly high prevalence of antibiotic use and excessive consumption, with both overuse and underuse patterns observed, underscoring the need for effective regulatory interventions and improved antibiotic stewardship in Yemen.

## Introduction

Antibiotics are a cornerstone of modern medicine, significantly reducing morbidity and mortality from bacterial infections [1]. However, their widespread and often inappropriate use has contributed to the alarming rise of antibiotic resistance, a phenomenon that the World Health Organization (WHO) and other global health authorities have identified as a critical public health threat [2–4].

This issue is particularly acute in hospital settings, where broad-spectrum antibiotics are frequently used to treat a wide range of infections, especially in critically ill patients [5,6]. This practice, while often necessary, fosters the emergence of multi-drug resistant organisms (MDROs), exacerbating the challenge of treating infections in these settings [7,8].

The problem is intensified by the global decline in the development of new antibiotics. Since the 1980s, the number of newly approved antibiotics has decreased sharply due to economic disincentives, regulatory challenges, and lower profitability compared to drugs for chronic conditions [9–12]. Consequently, the healthcare system remains heavily reliant on existing antibiotics, many of which are becoming less effective due to rising resistance levels.

In low- and middle-income countries (LMICs) like Yemen, antibiotic resistance poses an even greater challenge due to limited healthcare infrastructure, inadequate diagnostic capabilities, and poor regulation of antibiotic use [13,14]. The ongoing conflict in Yemen has further weakened the healthcare system, restricted access to laboratory diagnostics, and hindered the implementation of antibiotic stewardship programs. In tertiary hospitals in Sana’a, which serve critically ill patients, the lack of timely diagnostics often leads to empirical and prolonged use of broad-spectrum antibiotics, increasing the risk of resistance development [15–17].

Despite the urgency of the problem, there is a lack of local data on antibiotic use patterns in Yemen. This study addresses this gap by evaluating antibiotic consumption and prescribing practices in a tertiary hospital in Sana’a City. The findings aim to inform more effective antibiotic stewardship policies, particularly in resource-limited settings facing systemic challenges. As the first study of its kind in Yemen, it may also serve as a foundational model for monitoring and improving antibiotic use across similar healthcare settings in the region.

## Materials and methods

### Study population

The study population comprised adult patients admitted to the medical and surgical wards at a tertiary hospital. The wards were categorized into four distinct sections. Both male and female patients were included, covering a comprehensive range of general and private wards.

### Study design

This research was conducted as a prospective cross-sectional study, aiming to assess various parameters related to antibiotic use and resistance patterns among the hospitalized population. A cross-sectional design was chosen to provide a snapshot of antibiotic consumption practices at a specific point in time.

### Study location and period

The study was conducted at the University of Science and Technology Hospital (USTH) located in Sana’a, the capital city of Yemen. USTH is a teaching tertiary hospital established in 2005, recognized as one of the largest private hospitals in the region. The hospital has three critical care units: the Intensive Care Unit (ICU, 15 beds); the Cardiopulmonary Unit (CCU, 10 beds); and the Neurological Intensive Care Unit (NICU, 14 beds). The hospital has a total bed capacity of 190, with 104 beds allocated to the adult medical and surgical wards included in this study. Data were collected over a two-month period from January 12 to March 11, 2024. Although the study period was limited to two months, it included both weekdays and weekends and covered a representative patient flow. Moreover, no substantial seasonal variability in infectious disease prevalence was observed at the study site during the preceding year, making this period suitable for capturing typical prescribing patterns.

### Study sample

The final sample size (597 patients) was derived after applying the inclusion and exclusion criteria to the 1,381 patients screened. This approach aligns with WHO recommendations for point prevalence surveys in hospitals with fewer than 500 beds, where full enumeration is used rather than statistical sampling [18].

### Study data

Collected information included antibiotic daily dose strength (in grams), dosage form, frequency, start and end dates of antibiotic administration, admission and discharge dates, main diagnosis, serum creatinine levels, medical file number, patient age and gender, and discharge status (cure, discharge against medical advice [DAMA], discharge on request [DOR], transfer, or death).

Daily data collection was conducted manually from patient files and medication charts using a data collection form, typically between 8:00 AM and 2:00 PM, with occasional extensions to ensure all data were gathered. Data for patients discharged or admitted outside the standard collection hours were retrieved from the hospital’s Electronic Health Records (EHR).

Patients were grouped by age into four categories: young adult (18–25), adult (26–44), middle-aged (45–59), and old age (≥60). This is a globally accepted age classification [19].

### Inclusion and exclusion criteria

- **Inclusion Criteria:** Patients who received at least one systemic antibiotic, stayed for at least 24 hours, and had no requirement for dose adjustments (e.g., normal kidney function or specific cases of acute kidney injury [AKI]).
- **Exclusion Criteria:** Patients under 18 years old; those receiving only antifungal, antiviral, or anti-tuberculosis medications without antibiotics; patients discharged or transferred within 24 hours; patients with AKI requiring dose adjustment; and patients transferred into the included wards from ICU, CCU, NICU, or other hospitals, as their prior treatments or settings may confound assessment of initial empirical antibiotic use.

### Data preparation

Data were entered into a Microsoft Excel (2019) file, cross-verified, and then imported into SPSS for analysis.

### Enhancing data accuracy and minimizing bias

To enhance data accuracy and minimize biases, the following measures were taken:

- Daily review of EHRs to track the number of newly admitted patients.
- A double-check system in which two independent reviewers cross-verified the data entered into the Excel sheet against the original paper forms to identify and correct any errors. If discrepancies were found, they were resolved by rechecking the paper forms to ensure consistency with the recorded data.

### Measuring variables and metrics used

i **Defined Daily Dose (DDD):** The latest version of the WHO-ATC/DDD Index (2024 update) was used as a standardized measure for drug use comparison [20].
  - **Adjusted-DDD**: For this analysis, the DDDs for antibiotic combinations were adjusted to account for the total amount of both components, rather than just the primary antibiotic as assigned by the WHO. For example, while the WHO-DDD for parenteral Amoxicillin/Clavulanic acid is 3 grams based on Amoxicillin alone, in this analysis, it was adjusted to 3.6 grams to include the Clavulanic Acid component.
ii **Prescribed Daily Dose (PDD):** The PDD represents real-world prescribing patterns at the patient level. It was calculated by dividing the total grams consumed by the total patient-days for each patient individually; subsequently, the average of PDDs for all patients was calculated for each antibiotic separately.
iii **Patient-Days (PD):** Used to adjust antibiotic consumption for hospital activity, as it was found to be associated with the lowest overall assumption of wrong estimations compared to other metrics such as Days-Present (DP) or Days-of-Therapy (DOT).
iv **Consumption Density:** Measured as DDDs per 100 patient-days.
v **Deviation of PDD from DDD:** Categorized according to the percentage of deviation into: No-deviation (0%); Minor (< 5%); Moderate (≥ 5% and < 25%); or Major (≥ 25%) deviation [21]. The percentage of deviation (%) was calculated using this equation: [(PDD – DDD)/DDD] * 100 [22]. Positive deviation values indicate **overuse**, and negative values indicate **underuse**.

### Data analysis

Data were analyzed using SPSS version 27. Descriptive statistics were presented as medians with interquartile ranges (IQR) for continuous nonparametric variables and as percentages with absolute values for categorical variables. Bivariate correlation analysis explored relationships between variables; significant results were subjected to further analysis using crosstabs, Chi-Square, or Fisher’s Exact tests. A p-value of <.05 was considered statistically significant.

No formal correction (e.g., Bonferroni) was applied for multiple comparisons, as the study was exploratory in nature. However, p-values were interpreted cautiously, and findings were considered significant only when consistent with effect sizes and clinical relevance.

### Ethical considerations

The study maintained strict confidentiality of patient data. Patient consent was not required, as personal data were not collected. Ethical approval for the research (*No. LIU/UREC/2024/001*) was obtained from the University Research Ethics Committee (UREC) of Lebanese International University (LIU). Permission for data collection was obtained from the medical administration of USTH. The anonymized dataset shared as supplementary material (S1 File) does not contain any personally identifiable information and is shared in accordance with ethical approval.

## Results

**Fig 1:**
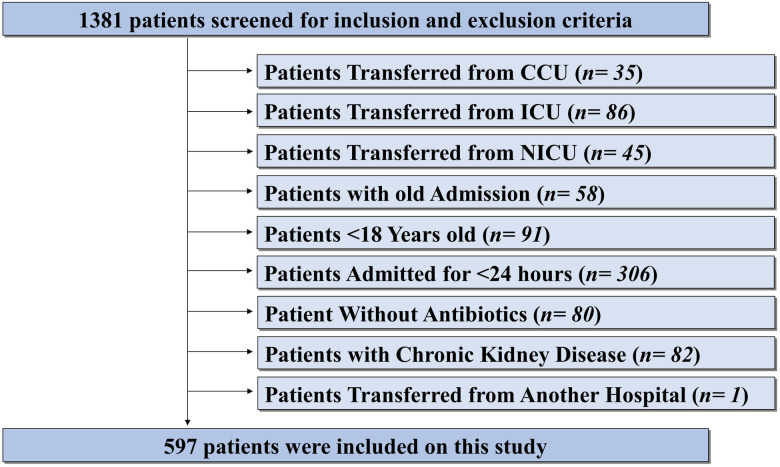
Case Selection Flowchart of Participants.

A total of 1,381 patients were screened in the study, which was conducted across four different medical and surgical wards. Of these, 597 patients, accounting for 1,504 patient-days, were included. Demographic and clinical characteristics are summarized in Tables 1 and 2, and the patient selection process is illustrated in Fig 1. The median age of patients was 38 years (IQR: 29–56.5, range: 18–96), with 45% aged between 26 and 45 years. Females comprised 64% of the patient population, and 55.1% were admitted to private wards.

**Table 1:** Demographic Characteristics of Patients (n = 597).

Characteristics		n	%	Median (IQR)	Min – Max
**Age (Years)**		597		38 (29-56.5)	18 - 96
**Age Groups**	**(18–25 age)**	108	18.1		
	**(26–44 age)**	266	44.6		
	**(45–59 age)**	88	14.7		
	**(≥60 age)**	135	22.6		
**Gender**	**Males**	217	36		
	**Females**	380	64		
**LOS**		597		2 (1-3)	1 - 15
	**1 to 2 days**	377	63.1		
	**3 to 6 days**	198	33.2		
	**≥ 7 days**	22	3.7		
**Discharge Status**	**Discharged by Doctor’s Order**	555	93		
	**DAMA or DOR**	26	4		
	**Transfer to ICU**	12	2		
	**Death**	4	1		
**Wards**	**Male Private**	124	20.8		
	**Male General**	95	15.9		
	**Female Private**	205	34.3		
	**Female General**	173	29.0		

DAMA: Discharged Against Medical Advice; DOR: Discharged on Request; ICU: Intensive Care Unit; LOS: Length of Hospital Stay.

**Table 2:** Clinical Characteristics of Included Patients.

Characteristics		n	%	Median (IQR)
**Serum Creatinine** ^**a**^ **(mg/dl)**		292	48.9	.78 (.6 − 1)
**Diagnosis** ^b^	**Infectious or Parasitic Diseases**	11	1.8	
	**Neoplasms**	18	3.0	
	**Blood or Blood-Forming Organs**	8	1.3	
	**Digestive System**	43	7.2	
	**Endocrine, Nutritional or Metabolic Diseases**	4	.7	
	**Circulatory System**	25	4.2	
	**Musculoskeletal System or Connective Tissue**	53	8.9	
	**Nervous System**	11	1.8	
	**External Causes of Morbidity or Mortality**	11	1.8	
	**Respiratory System**	65	10.9	
	**Pregnancy, childbirth or the puerperium**	153	25.6	
	**Genitourinary System**	22	3.7	
	**Diseases of Skin**	8	1.3	
	**Post-Surgical/Endoscopic Procedures**	160	26.8	
	**Others** ^**c**^	5	.8	
**Infection Diagnosis**	**Yes**	127	21.3	
	**No**	470	78.7	
**Antibiotics Indication** **Type**	**Empirical**	127	21.3	
	**Post-Surgical**	295	49.4	
	**Medical Prophylaxis**	19	3.2	
	**No Clear indication**	156	26.1	

^a^Serum creatinine values were only recorded if available in the patient chart. No imputation was performed for missing values. Since renal-impaired patients requiring dose adjustments were excluded, the missing data were not expected to bias the findings related to antibiotic consumption.

^a^Diagnosis categorization is based on International Classification of Diseases ICD-11 [23].

^c^Other diseases include: Edema (n = 1), headache (n = 1), Malaise & fatigue (n = 1) and Immune system (n = 2).

The median length of hospital stay (LOS) was 2 days (IQR: 1–3). Approximately 63% of patients were hospitalized for less than 3 days, and 93% were discharged per physician’s orders. During the study period, 2% (12 cases) were transferred to the ICU, and 1% (4 cases) died. The median serum creatinine level was.78 mg/dL (IQR:.6–1.0 mg/dL) as patients with chronic kidney disease were excluded. The most common diagnoses were “Post-Surgical or Post-Endoscopic Procedures” (26.8%), followed by “Pregnancy, childbirth or the puerperium” (25.6%). The proportion of patients diagnosed with an infection was 21.3%, all of whom received empirical antibiotic therapy. The most common indications for antibiotic use were post-surgical operations (49.4%), specifically prolonged surgical prophylaxis (>24 hours), followed by prescriptions without a documented indication (26.1%). Antibiotics prescribed for medical prophylaxis accounted for 3.2% of cases.

### Antibiotic prescription patterns

Thirty different antibiotics were prescribed during the study period. Of these, 17 agents (56.7%) were from the “Watch” category of the WHO-AWaRe classification system, nine (30%) from the “Access” category, and the remainder from the “Reserve” (6.7%) and “Not Recommended” (6.7%) categories. Approximately 40% of prescribed antibiotics were from the beta-lactam group, which accounted for 50% of all prescriptions, followed by fluoroquinolones at 10% of prescribed agents and 10.2% of prescriptions.

The most commonly prescribed antibiotics, representing 75% of drug utilization (DU75), were Ceftriaxone, Metronidazole, Vancomycin, Levofloxacin, Imipenem/Cilastatin, and Moxifloxacin. Ceftriaxone was prescribed 380 times (33.6%) with a DDD/100 patient-days of 51 (63 with STAT orders). Metronidazole was prescribed 247 times (21.8%), with a DDD/100 patient-days of 29 (33 with STAT orders). Vancomycin (*n* = 68; 6.0%), Levofloxacin (*n* = 55; 4.9%), Imipenem/Cilastatin (*n* = 53; 4.7%), and Moxifloxacin (*n* = 41; 3.6%) had DDD/100 patient-days of 12.7, 12.4, 6.8, and 8.5, respectively (Tables 3 and 4).

**Table 3:** Descriptive Statistics of all Prescribed Antibiotics (n = 30).

Antibiotic Groups *n* = 13	Antibiotics *n* = 30	WHO ATC Code	WHO AWaRe Class	Prescriptions (*n* = 1132)		DDD/100 Patient-Days
				*n* (%)	Grams	
**Beta-lactams**	Ceftriaxone Course	J01DD04	Watch	380 (33.57)	1533	50.96
	Course + STAT				1719	63.33
	Piperacillin/tazobactam	J01CR05	Watch	33 (2.92)	1122	4.74
	Cefuroxime	J01DC02	Watch	29 (2.56)	156	3.46
	Cefepime	J01DE01	Watch	27 (2.39)	105	1.75
	Amoxicillin/Clavulanic acid	J01CR02	Access	24 (2.12)	160.8	2.97
	Cefpirome	J01DE02	Watch	21 (1.86)	120	1.99
	Ampicillin/Sulbactam	J01CR01	Access	19 (1.68)	108	.80
	Cefoperazone	J01DD12	Watch	19 (1.68)	71	1.18
	Cefoperazone/Sulbactam	J01DD62	Not recom	10 (.88)	60	.66
	Cefixime	J01DD08	Watch	1 (.09)	.8	.13
	Ceftazidime	J01DD02	Watch	1 (.09)	4	.07
	Ceftriaxone+Sulbactam	J01DD63	Not recom	1 (.09)	6	.13
**Total (%)**	12 (40.0%)			565 (49.90)		
**Fluoroquinolones**	Levofloxacin	J01MA12	Watch	55 (4.86)	93.5	12.43
	Moxifloxacin	J01MA14	Watch	41 (3.62)	51.2	8.51
	Ciprofloxacin	J01MA02	Watch	19 (1.68)	14.8	1.23
**Total (%)**	3 (10.0%)			115 (10.20)		
**Carbapenems**	Imipenem/cilastatin	J01DH51	Watch	53 (4.68)	411	6.83
	Meropenem	J01DH02	Watch	32 (2.83)	242.5	5.60
**Total (%)**	2 (6.7%)			85 (7.5)		
**Glycopeptides**	Vancomycin	J01XA01	Watch	68 (6.01)	381.5	12.68
**Total (%)**	1 (3.3%)			68 (6)		
**Aminoglycosides**	Gentamicin	J01GB03	Access	28 (2.47)	10.1	2.80
	Amikacin	J01GB06	Access	2 (.18)	11	.73
**Total (%)**	2 (6.7%)			30 (2.70)		
**Tetracyclines**	Doxycycline	J01AA02	Access	4 (.35)	1.9	1.26
**Total (%)**	1 (3.3%)			4 (.40)		
**Lincosamides**	Clindamycin	J01FF01	Access	4 (.35)	19.44	.72
**Total (%)**	1 (3.3%)			4 (.40)		
**Oxazolidinones**	Linezolid	J01XX08	**Reserve**	2 (.18)	6	.33
**Total (%)**	1 (3.3%)			2 (.20)		
**Nitroimidazoles**	Metronidazole Course	J01XD01	Access	247 (21.82)	660.5	29.28
	Course + STAT				721.5	33.33
	Ornidazole	P01AB03	Access	2 (.18)	4.5	.20
**Total (%)**	2 (6.7%)			249 (22)		
**Macrolides**	Clarithromycin	J01FA09	Watch	2 (.18)	3.5	.47
	Erythromycin	J01FA01	Watch	1 (.09)	1.5	.10
**Total (%)**	2 (6.7%)			3 (.30)		
**Rifamycins**	Rifaximin	A07AA11	Watch	1 (.09)	4.4	.49
**Total (%)**	1 (3.3%)			1 (.10)		
**Glycylcyclines**	Tigecycline	J01AA12	**Reserve**	2 (.18)	1.65	1.10
**Total (%)**	1 (3.3%)			1 (.20)		
**Sulfonamides**	Trimethoprim/Sulfamethoxazole	J01EE01	Access	4 (.35)	12.96	.45
**Total (%)**	1 (3.3%)			1 (.40)		

ATC = anatomical, therapeutical, and chemical; Not recom = Not recommended; WHO = World Health Organization. STAT doses: are the doses given outside of admission wards.

Note: Overall, 56.7% of prescribed antibiotics were from the ‘**Watch’** category.

**Table 4:** Descriptive Statistics of Antibiotics with ≥10 Prescriptions for PDD-DDD Deviation.

Antibiotics	Overuse	Underuse	Normal Dose	Total Prescriptions
	n (%)	n (%)	n (%)	n
**Ceftriaxone Course**	114 (30.0)	59 (15.5)	207 (54.5)	380
**Ceftriaxone Course + STAT**	197 (51.8)	28 (7.4)	155 (40.8)	380
**Metronidazole Course**	62 (25.1)	111 (44.9)	74 (30.0)	247
**Metronidazole Course + STAT**	87 (35.2)	80 (32.4)	80 (32.4)	247
**Vancomycin**	19 (27.9)	14 (20.6)	35 (51.5)	68
**Levofloxacin**	26 (47.3)	0 (.0)	29 (52.7)	55
**Imipenem/Cilastatin**	2 (3.8)	51 (96.2)	0 (.0)	53
**Moxifloxacin**	22 (53.7)	0 (.0)	19 (46.3)	41
**Piperacillin/Tazobactam**	8 (24.2)	20 (60.6)	5 (15.2)	33
**Meropenem**	5 (15.6)	18 (56.3)	9 (28.1)	32
**Cefuroxime**	8 (27.6)	14 (48.3)	7 (24.1)	29
**Gentamicin**	4 (14.3)	11 (39.3)	13 (46.4)	28
**Cefepime**	0 (.0)	25 (92.6)	2 (7.4)	27
**Amoxicillin/Clavulanic acid**	4 (16.7)	15 (62.5)	5 (20.8)	24
**Cefpirome**	0 (.0)	19 (90.5)	2 (9.5)	21
**Ampicillin/Sulbactam**	0 (.0)	19 (100.0)	0 (.0)	19
**Ciprofloxacin**	3 (15.8)	16 (84.2)	0 (.0)	19
**Cefoperazone**	0 (.0)	19 (100.0)	0 (.0)	19
**Cefoperazone/Sulbactam**	0 (.0)	9 (90.0)	1 (10.0)	10

**STAT doses**: are the doses given outside of admission wards.

### Prevalence of antibiotics use

The prevalence of antibiotic prescriptions among included patients was 92.5%. This prevalence was calculated by excluding patients discharged within 24 hours, even if they received antibiotics.

### Discrepancies in antibiotic consumption

Discrepancies were observed between the average PDD and the WHO-DDD. Of the 30 antibiotics (plus 2 repeated with STAT doses), only those with 10 or more prescriptions (n = 19) were included in the deviation analysis. Seven antibiotics (36.8%) were overused, while 12 (63.2%) were underused (Table 4).

Antibiotics were further classified by the extent of PDD deviation from DDD into major, moderate, or minor categories. One antibiotic (5.3%) was majorly overused, three (15.8%) were moderately overused, and three (15.8%) were minorly overused. Conversely, seven antibiotics (36.8%) were majorly underused, four (21.1%) were moderately underused, and one (5.3%) was minorly underused (Table 5).

**Table 5:** Number and Percentage of Antibiotics in Each Deviation Category.

Deviation Class	All Abs (*n = *30) +2	Abs With ≥ 10 Prescriptions (*n = *17) +2
**Major Overuse**	5 (15.63%)	1 (5.26%)
**Major Underuse**	11 (34.38%)	7 (36.84%)
**Moderate Overuse**	4 (12.50%)	3 (15.79%)
**Moderate Underuse**	5 (15.63%)	4 (21.05%)
**Minor Overuse**	3 (9.38%)	3 (15.79%)
**Minor Underuse**	1 (3.13%)	1 (5.26%)
**No Deviation**	3 (9.38%)	0 (.00%)

Abs = Antibiotics; + 2 indicates that ceftriaxone and metronidazole were each analyzed twice: once for course doses, and once for course doses combined with STAT doses.

### Analysis of antibiotics consumption

**Fig 2:**
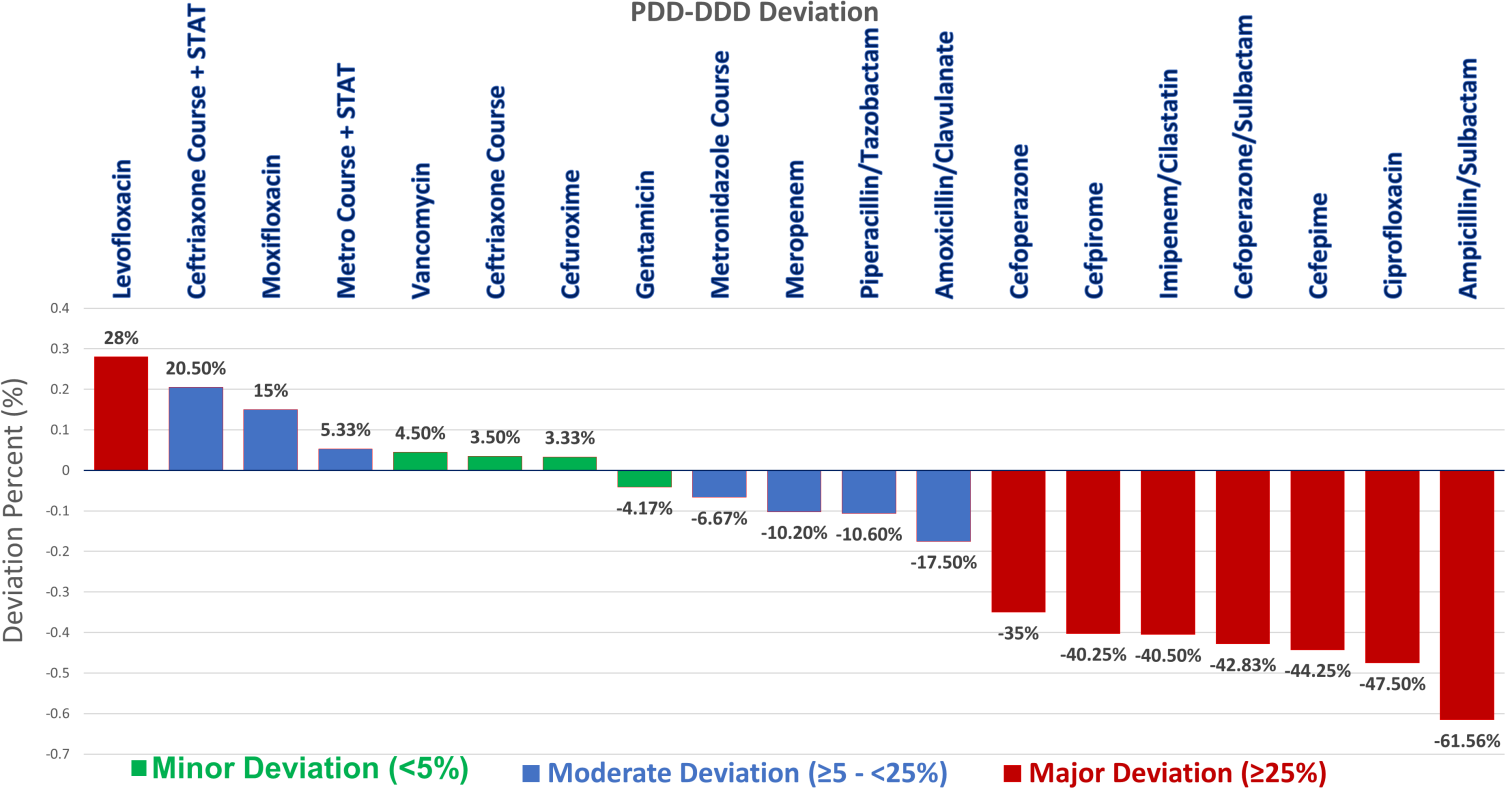
Deviation of mean PDD from DDD for antibiotics with ≥ 10 prescriptions: This figure illustrates the percentage deviation of mean PDD from WHO-DDD. Positive deviations (above the axis line) represent overuse, and negative deviations (below the line) represent underuse. Note: Bars represent % deviation of PDD from DDD. One-sample t-tests were used to assess significance.

A One-Sample t-test comparing mean PDD to DDD as a test value revealed significant deviations for several antibiotics (Fig 2 and Table 6).

**Table 6:** One-Sample T-Test for Mean PDD of Antibiotics with ≥10 Prescriptions.

Antibiotics	Test Values*	Mean (PDD) ^§^	*SD*	*t*	*df*	Sig. (2-tailed)	Mean Diff.	95% CI Lower; Upper	Cohen’s d
**Ceftriaxone**	2	2.07	.62	2.29	379	.022	.07	.01;.14	.62
**Course+STAT**		2.41	.71	11.39	379	<.001	.41	.34;.49	.49
**Metronidazole**	1.5	1.47	.57	−3.39	246	<.001	−.12	−.19; −.05	.57
**Course+STAT**		1.55	.54	1.56	246	.120	.05	−.01;.12	.12
**Vancomycin**	2	2.09	.76	1.00	67	.322	.09	−.09;.28	.76
**Levofloxacin**	.5	.64	.21	5.01	54	<.001	.14	.08;.19	.21
**Imipenem/Cilastatin**	4(2)^**†**^	2.38	.97	−12.13	52	<.001	−1.62	−1.88; −1.35	.97
**Moxifloxacin**	.4	.46	.10	3.47	40	.001	.06	.02;.09	.10
**Piperacillin/Tazobactam**	15.75(14)^**†**^	14.10	5.3	−1.83	32	.077	−1.67	−3.53;.19	5.25
**Meropenem**	3	2.70	1.2	−.57	31	.574	−.12	−.56;.32	1.22
**Cefuroxime**	3	3.10	1.7	.32	28	.752	.10	−.54;.74	1.67
**Gentamicin**	.24	.23	.08	−.93	27	.361	−.01	−.05;.02	.08
**Cefepime**	4	2.23	.63	−14.62	26	<.001	−1.77	−2.01; −1.52	.63
**Amoxicillin/Clavulanic acid**	3.6(3)^**†**^	2.97	.91	−3.38	23	.003	−.63	−1.01; −.24	.91
**Cefpirome**	4	2.39	.78	−9.49	20	<.001	−1.61	−1.97; −1.26	.78
**Ampicillin/Sulbactam**	9(6)^**†**^	3.46	1.25	−19.26	18	<.001	−5.54	−6.14; −4.94	1.25
**Ciprofloxacin**	.8	.52	.23	−5.30	18	<.001	−.28	−.4; −.17	.23
**Cefoperazone**	4	2.10	.45	−18.34	18	<.001	−1.90	−2.12; −1.69	.45
**Cefoperazone/Sulbactam**	6(4)^**†**^	3.43	1.1	−7.36	9	<.001	−2.58	−3.37; −1.78	1.11

*The test values represent the WHO-DDD. ^§^The mean values represent the estimated PDD.

^†^The values used are the Adjusted-DDDs which include the second component of antibiotic combinations, as the WHO-DDDs (values between brackets) were assigned for main component only.

Note: One-sample t-test was used to compare PDD to WHO-DDD values.

Levofloxacin showed major overuse, with a 28% deviation from the DDD (*M* = .64 ± .21). The one-sample t-test indicated a highly significant difference *t*(54) = 5.01, *p* < .001, with a mean difference of.14 grams (95% *CI*:.08 to.19). The effect size (Cohen’s *d* = .21) indicated a small effect.

Moxifloxacin was moderately overused, deviating by 15% (*M* = .46 ± .10), *t*(40) = 3.47, *p* < .001, with a mean difference of.06 grams (95% *CI*:.02 to.09), indicating a highly significant difference. The effect size (Cohen’s *d* = .1) indicated a small effect.

Ceftriaxone was significantly overused with a minor deviation of 3.5% (*M* = 2.07 ± .62), *t*(379) = 2.29, *p* = .022, with a mean difference of.07 grams (95% *CI*:.01 to.14). The effect size (Cohen’s *d* = .62) indicated a medium effect. Although this deviation was significantly increased to 20.5% with STAT orders, categorizing it as moderately overused (*M* = 2.41 ± .71), *t*(379) = 11.39, *p* < .001, with a mean difference of.41 grams (95% *CI*:.34 to.49). The Cohen’s *d* = .71 indicated a medium to large size effect.

Metronidazole was moderately underused with a significant deviation of −6.67% (*M* = 1.47 ± .57), *t*(246) = 3.39, p < .001, with a mean difference of −.12 grams (95% *CI*: −.19 to −.05). The effect size (Cohen’s d = 0.57) indicated a medium effect. Although this deviation shifted to moderate overuse (5.33%) when including STAT doses, albeit without statistical significance (*M* = 1.55 ± .54), *t*(246) = 1.56, *p* = .12.

The most significant deviations were observed in the majorly underused category. Imipenem/Cilastatin had a mean PDD that was 40.5% lower than the DDD, classifying it as majorly underused, indicating a highly significant difference (*M* = 2.38 ± .97), *t*(52) = 12.13, *p* < .001, with a mean difference of −1.62 grams (95% *CI*: −1.88 to −1.35). The effect size (Cohen’s *d* = .97) indicated a large effect.

Similar underuse was noted for Cefepime (*M* = 2.23 ± .63), *t*(26) = 14.62, *p* < .001, Cefpirome (*M* = 2.39 ± .78), *t*(20) = 9.49, *p* < .001, Ampicillin/Sulbac*t*am (*M* = 3.46 ± 1.25), *t*(18) = 19.26, *p* < .001, and Ciprofloxacin (*M* = .52 ± .23), *t*(18) = 5.30, *p* < .001, wi*t*h deviations of −44.3%, −40.3%, −61.6%, and −47.5%, respec*t*ively. Cefoperazone (*M* = 2.10 ± .45) and Cefoperazone/Sulbactam (*M* = 3.43 ± 1.11) were also majorly underused with deviations of −35%, *t*(18) = 18.34, *p* < .001, and −42.8%, *t*(9) = 7.36, *p* < .001, respectively.

In the moderately underused category, Amoxicillin/Clavulanic acid (*M* = 2.97 ± .91), Piperacillin/Tazobactam (*M* = 14.10 ± 5.3), and Meropenem (*M* = 2.70 ± 1.22) showed deviations from DDD by −17.5%, *t*(23) = 3.38, *p* = .003, −10.6%, *t*(32) = 1.83, *p* = .077, and −10.2%, *t*(31) =.57, *p* = .57, respectively; however, the latter two results were not statistically significant.

Antibiotics classified under minor deviations, such as Vancomycin, Cefuroxime, and Gentamicin, were considered to be consistent with DDDs, as the percentage of deviation was minimal, and the T-Test did not show significant deviations (Table 6).

### Factors associated with antibiotics deviation

A Pearson Chi-Square crosstabulation test was conducted to evaluate the association between antibiotic deviation and independent variables. Significant associations were found between the deviation of some antibiotics and age, gender, and ward type.

**Ceftriaxone**: A significant association was observed between age group and deviation of Ceftriaxone PDD with STAT doses from DDD, *X*^*2*^(6, **N* *= 380) = 30.34, *p* < .001. Among patients who received overused doses, 57.9% were between 26 and 45 years old. A similar association was found between the type of ward and Ceftriaxone deviation from DDD, *X*^*2*^(6, **N* *= 380) = 40.56, *p* < .001, with 47.7% of patients receiving overused doses in female private ward. Gender also significantly influenced the deviation of Ceftriaxone from DDD, *X*^*2*^(2, **N* *= 380) = 35.17, *p* < .001, with females receiving a higher proportion of overused doses (83.2%) compared to males (16.8%) (Table 7).

**Table 7:** Association Between (Age, Gender, and Ward) and Ceftriaxone Consumption.

		Ceftriaxone Use				Pearson Chi-Square		
		Overuse	Underuse	Normal Dose	Total			
		*n* (%)	*n* (%)	*n* (%)	*n* (%)	Value	*df*	*p*
**Age Groups (Years)**	**18-25**	47 (23.9)	4 (14.3)	28 (18.1)	79 (20.8)	30.3	6	<.001
	**26-44**	**114 (57.9)**	8 (28.6)	65 (41.9)	187 (49.2)			
	**45-59**	14 (7.1)	7 (25.0)	25 (16.1)	46 (12.1)			
	**≥60**	22 (11.2)	9 (32.1)	37 (23.9)	68 (17.9)			
	**Total**	197 (100)	28 (100)	155 (100)	380 (100)			
**Gender**	**Male**	33 (16.8)	17 (60.7)	60 (38.7)	110 (28.9)	35.2	2	<.001
	**Female**	**164 (83.2)**	11 (39.3)	95 (61.3)	270 (71.1)			
	**Total**	197 (100)	28 (100)	155 (100)	380 (100)			
**Ward**	**M.Pri**	17 (8.6)	11 (39.3)	32 (20.6)	60 (15.8)	40.6	6	<.001
	**M.Gen**	17 (8.6)	6 (21.4)	29 (18.7)	52 (13.7)			
	**F.Pri**	**94 (47.7)**	8 (28.6)	43 (27.7)	145 (38.2)			
	**F.Gen**	69 (35.0)	3 (10.7)	51 (32.9)	123 (32.4)			
	**Total**	197 (100)	28 (100)	155 (100)	380 (100)			

M. Pri = Male private; M.Gen = Male general; F.Pri = Female private; F.Gen = Female general.

The significant difference related to gender suggests that female patients are more susceptible to receiving overdoses of Ceftriaxone, potentially due to differences in dispensing or prescribing practices by nurses and physicians. This difference underscores the need for more tailored antibiotic dosing guidelines that consider gender.

**Metronidazole**: The PDD-DDD deviation of Metronidazole was significantly influenced by independent variables such as age and gender (Table 8). The most substantial deviations in the Metronidazole course occurred among patients aged 26–44 years, with [(53.2% Overuse) and (55.0% Underuse] class deviations found in this age group, *X²* (6, **N* *= 247) = 13.07, *p* = .042. Gender also significantly influenced the deviation of Metronidazole PDD from DDD. Among the 173 patients who received deviated doses, 82.7% were female, with approximately 87.4% of the underused doses found among female group, *X²* (2, **N* *= 247) = 9.0, *p* = .011. The association of gender with deviated doses could be due to the differences in clinical decision-making processes. Addressing these differences is crucial for optimizing treatment outcomes and preventing antibiotic misuse in female patients.

**Table 8:** Association Between (Age and Gender) and Metronidazole Consumption.

		Metronidazole Use				Pearson Chi-Square		
		Overuse	Underuse	Normal Dose	Total			
		*n* (%)	*n* (%)	*n* (%)	*n* (%)	Value	*df*	*p*
**Age Groups** **(Years)**	**18-25**	9 (14.5)	33 (29.7)	18 (24.3)	60 (24.3)	13.1	6	.042
	**26-44**	**33 (53.2)**	**61 (55.0)**	34 (45.9)	128 (51.8)			
	**45-59**	8 (12.9)	7 (6.3)	13 (17.6)	28 (11.3)			
	**≥60**	12 (19.4)	10 (9.0)	9 (12.2)	31 (12.6)			
	**Total**	62 (100)	111 (100)	74 (100)	247 (100)			
**Gender**	**Male**	16 (25.8)	14 (12.6)	22 (29.7)	52 (21.1)	9.0	2	.011
	**Female**	**46 (74.2)**	**97 (87.4)**	52 (70.3)	195 (78.9)			
	**Total**	62 (100)	111 (100)	74 (100)	247 (100)			

### Impact of antibiotics consumption on LOS and discharge status

A crosstabulation with Chi-Square analysis was performed to assess the impact of antibiotics with more than 25 prescriptions on discharge status and LOS. Significant findings were observed for Ceftriaxone, Vancomycin, Amoxicillin/Clavulanic acid, and Gentamicin.

**Ceftriaxone**: A significant association was found between the deviation of Ceftriaxone doses and discharge status, *X²* (1, **N* *= 380) = 9.55, *p* = .002. Among patients who were discharged without a doctor’s order (e.g., DAMA, DOR, death, or ICU transfer), 74.1% received deviated doses. Specifically, 57.1% of DAMA patients, 83.3% of transferred cases, and all patients who either died or were discharged on request received deviated doses of Ceftriaxone (Fisher’s Exact test *2-sided*, p = .005) (Table 9).

**Table 9:** Impact of Ceftriaxone Consumption on Discharge Status.

		Ceftriaxone Dosing			Value	*df*	*P* ^*c*^
		Deviated Dose n (%)	Normal Dose n (%)	Total n (%)			
**Discharged by Dr Order**	**Yes**	153 (88.4)	200 (96.6)	353 (92.9)	9.55	1	.002^a^
	**No**	**20 (11.6)**	**7 (3.4)**	**27 (7.1)**			
	**Total**	173 (100)	207 (100)	380 (100)			
**Discharge Status**	**By Dr Order**	153 (88.4)	200 (96.6)	353 (92.9)	12.54	4	.005^b^
	**DAMA**	8 (4.6)	6 (2.9)	14 (3.7)			
	**Transfer to ICU**	5 (2.9)	1 (.5)	6 (1.6)			
	**Death**	2 (1.2)	0 (.0)	2 (.5)			
	**DOR**	5 (2.9)	0 (.0)	5 (1.3)			
	**Total**	173 (100)	207 (100)	380 (100)			

^a^Pearson Chi-Square; b. Fisher’s Exact Test; c. Exact Sig. (2-sided) P-Values.

The deviation of Ceftriaxone doses, particularly with STAT orders, showed a significant association with LOS, *X²* (2, **N* *= 380) = 17.50, *p* < .001. Paradoxically, the LOS for patients who received overdoses (90.9%), especially in the major overuse class (93.7%), was 1–3 days. Additionally, patients who received normal doses were more likely to stay longer than three days (63.5%), compared to those who received overdoses (28.6%) or underdoses (7.9%) (Table 10).

**Table 10:** Impact of Antibiotics Misuse on Length of Hospital Stays (LOS).

Antibiotics	Group-Variables	Length of Hospital Stays			Value	*df*	*P* ^*c*^
		1-3 Days	> 3 Days	Total			
		*n* (%)	*n* (%)	*n* (%)			
**Ceftriaxone W STAT Use**	**Overuse**	179 (56.5)	18 (28.6)	197 (51.8)	17.50	2	<.001^a^
	**Underuse**	23 (7.3)	5 (7.9)	28 (7.4)			
	**Normal Dose**	115 (36.3)	40 (63.5)	155 (40.8)			
	**Total**	317 (100.0)	63 (100.0)	380 (100.0)			
**Ceftriaxone W STAT Deviation Categories** ^**†**^	**Moderate+**	15 (4.7)	7 (11.1)	22 (5.8)	29.02	4	<.001^b^
	**Major+**	164 (51.7)	11 (17.5)	175 (46.1)			
	**Moderate-**	3 (.9)	1 (1.6)	4 (1.1)			
	**Major-**	20 (6.3)	4 (6.3)	24 (6.3)			
	**Normal**	115 (36.3)	40 (63.5)	155 (40.8)			
	**Total**	317 (100.0)	63 (100.0)	380 (100.0)			
**Amoxicillin/Clavulanic Acid Use**	**Overuse**	1 (5.6)	3 (50.0)	4 (16.7)	5.75	2	.033^b^
	**Underuse**	12 (66.7)	3 (50.0)	15 (62.5)			
	**Normal Dose**	5 (27.8)	0 (.0)	5 (20.8)			
	**Total**	18 (100.0)	6 (100.0)	24 (100.0)			
**Vancomycin Use**	**Overuse**	13 (76.5)	6 (37.5)	19 (57.6)	5.16	1	.024^a^
	**Underuse**	4 (23.5)	10 (62.5)	14 (42.4)			
	**Total**	17 (100.0)	16 (100.0)	33 (100.0)			
**Vancomycin Deviation Categories** ^**†**^	**Moderate+**	1 (2.6)	4 (13.8)	5 (7.4)	12.46	4	.008^b^
	**Major+**	12 (30.8)	2 (6.9)	14 (20.6)			
	**Moderate-**	1 (2.6)	2 (6.9)	3 (4.4)			
	**Major-**	3 (7.7)	8 (27.6)	11 (16.2)			
	**Normal Dose**	22 (56.4)	13 (44.8)	35 (51.5)			
	**Total**	39 (100.0)	29 (100.0)	68 (100.0)			
**Gentamicin Deviation Categories** ^**†**^	**Moderate+**	0 (.0)	1 (25.0)	1 (3.6)	7.70	3	.029^b^
	**Major+**	3 (12.5)	0 (.0)	3 (10.7)			
	**Major-**	8 (33.3)	3 (75.0)	11 (39.3)			
	**Normal Dose**	13 (54.2)	0 (.0)	13 (46.4)			
	**Total**	24 (100.0)	4 (100.0)	28 (100.0)			

^a^Pearson Chi-Square; ^b^. Fisher’s Exact Test; ^c^. Exact Sig. (2-sided) P-Values.

^†^“+” = Overuse; “-” = Underuse.

**Amoxicillin/Clavulanic acid**: More than three-quarters of patients received deviated doses, with 58.3% of deviations in the major underuse class. The association between Amoxicillin/Clavulanic acid consumption and LOS was significant. Nearly 100% of those who received normal doses and 80% of those in the underuse class were discharged within 1–3 days, compared to 25% in the overuse class. Moreover, 75% of patients in the overuse class had a hospital stay exceeding three days (Fisher’s Exact test, *p* = .033) (Table 10).

**Vancomycin**: Vancomycin underuse also significantly associated with longer LOS. Similar to Ceftriaxone, the LOS for patients who received overdoses (68.4%) was 1–3 days, while 71.4% of patients in the underuse class stayed longer than three days, *X²* (1, **N* *= 33) = 5.16, *p* = .024. Additionally, 44.8% of patients who had a LOS of more than 3 days received non-deviated doses, (Fisher’s Exact test, *p* = .008, (Table 10).

**Gentamicin**: Deviated Gentamicin consumption significantly associated with longer LOS, *X²* (3, **N* *= 28) = 10.182, *p* = .017 (Table 10). Nearly 100% of patients with non-deviated doses were discharged within 1–3 days, whereas 75% of those who stayed longer than three days had received majorly underused doses of Gentamicin (Fisher’s Exact test, *p* = .029), (Table 10).

## Discussion

This study is the first in Yemen to evaluate antibiotic consumption and use specifically in hospital wards, rather than ICU settings. Key findings include a high prevalence of antibiotic use, particularly for surgical prophylaxis; notable consumption of beta-lactams, especially third-generation cephalosporins like Ceftriaxone; significant deviations from recommended antibiotic dosages; notable associations between antibiotic misuse and outcomes, including longer hospital stays; and an emphasized need for strong stewardship policies to guide appropriate antibiotic use.

This study found a notably high prevalence of antibiotic use (92.5%) among hospitalized patients, despite only 21.3% having an infection diagnosis. This is particularly concerning given that the study was conducted in non-ICU settings, which typically have a lower need for antibiotic prescriptions. This prevalence is higher than that reported in 29 European countries, global data from 53 countries, and studies from Africa and Thailand, where the reported prevalence was found to be 30.5%, 34.4%, 56%, and 53%, respectively [6,24–26]. About half of patients received antibiotics after surgical operations, and their use extended until the end of hospital stays in most cases. This finding mirrors findings from Thailand [26], but contrasts with global guidelines that limit prophylaxis to 24 hours and rarely to 48 hours in some surgery types [27]. Extending the duration of antibiotic prophylaxis may result in poor long-term outcomes, as reported in a multinational study including the United States, Canada, Australia, Norway, and India. They reported that patients with open fractures and mild contamination who received an extended duration of antibiotic prophylaxis had a higher risk of developing deep soft tissue infections compared to those with a shorter duration [28]. The high prevalence of antibiotic use and the extended duration of antibiotic administration indicate a misuse of these life-saving agents, underscoring the immediate need for clinical interventions and regulatory policies to minimize this misuse.

Most prescribed antibiotics (56.67%) were from the WHO’s “Watch” category, with only 30% from the “Access” category, indicating a preference for broad-spectrum antibiotics like third-generation cephalosporins, quinolones, and carbapenems. Similar trends have been observed in other LMICs like Ethiopia, India, and Pakistan, highlighting the regional challenge of over-reliance on broad-spectrum antibiotics [29–31]. The beta-lactam group, particularly Ceftriaxone and Metronidazole, were the most prescribed, consistent with other studies from Yemen, India, and the Middle East [6,32–35]. This pattern is inconsistent with WHO recommendations, which advocate for “Access” antibiotics as first-line treatments to reduce resistance [36], and with global trends where the “Access” category represents 50% of antibiotic consumption [37].

This pattern emphasizes the need for implementing antibiotic stewardship programs and rapid diagnostic tests to limit broad-spectrum antibiotic use, which is often driven by the desire for rapid clinical outcomes and by delayed diagnostic results [17,38,39].

The study also revealed significant deviations between PDD and DDD, with both overuse and underuse observed. For instance, Levofloxacin was overused by 28%, while Imipenem/Cilastatin was underused by −40.5%. Similar findings were reported in other studies where patterns of overuse and underuse were encountered [22]. Although DDDs do not always perfectly align with actual PDD, the substantial deviations are concerning, particularly in the majorly underused class, as PDDs were noted to be inconsistent with dosing guidelines in many prescriptions, which places antibiotics at a high risk of resistance development [14]. For instance, Ciprofloxacin was prescribed at 0.4 g/day, while the minimum recommended dose is 0.8 g/day. Also, Ampicillin/Sulbactam was underused at 3.46 g/day, and Imipenem/Cilastatin was underdosed at 2.4 g/day, while the minimum recommended doses are 6 g/day and 2.4 g/day, respectively. This high deviation, particularly for combined antibiotics, may reflect a misunderstanding of dosing guidelines, which typically focus on the main component alone without accounting for the second component [40]. These discrepancies highlight the need for regular training and education for healthcare providers on appropriate antibiotic dosing. Additionally, establishing protocols for empirical therapy based on local susceptibility patterns can help reduce inappropriate antibiotic use.

The study identified significant associations between patient demographics (age, gender, and ward) and deviations in PDD from DDD. Notably, adult patients and females, especially in the female private ward, were more likely to receive deviated doses of Ceftriaxone and Metronidazole. This contrasts with other studies, where older males were more prone to antibiotic misuse [34,41,42]. The age difference might result from this study’s exclusion of elderly patients with kidney failure, who were included in other studies; however, the observed gender differences indicate potential disparities in prescribing practices, highlighting the need for further research into gender-specific dosing guidelines.

Deviations in antibiotic dosing were significantly associated with patient outcomes, including LOS and discharge status. Patients receiving overused doses of Ceftriaxone had a shorter LOS, typically between 1–3 days, compared to those who received normal doses. This counterintuitive finding may suggest that overdose could lead to quicker discharge, but possibly at the expense of long-term health outcomes, or it may indicate a change in the minimum inhibitory concentration (MIC) due to overuse. This finding was reflected in other studies in which the MIC of Ceftriaxone was found to be higher than the standard value of the Clinical & Laboratory Standards Institute (CLSI) [7,43,44].

### Study limitations and strengths

This study has some limitations that need to be acknowledged:

a **Single-center design:** The findings may not be generalizable to other hospitals in Yemen or internationally due to differences in healthcare systems and antibiotic prescribing practices. However, antibiotic prescribing patterns in Sana’a are likely similar across hospitals, as many physicians work in multiple settings and share practices.
b **Inherent PDD vs. DDD discrepancy**: Comparing PDD with WHO-DDD is inherently limited, as DDD does not account for patient-specific factors like disease severity or comorbidities. While the study excluded pediatric patients, those in the ICU, and patients with renal or hepatic impairment to reduce this discrepancy, the observed simplicity of infection in wards and significant deviations in antibiotic use justify this limitation.
c **Manual data collection**: Manual data collection may lead to bias, but steps were taken to mitigate this, such as cross-checking by independent reviewers.
d **Retrieving data from EHR:** Retrieving data from EHR may introduce recall bias, particularly if any information was missing and had to be supplemented by nursing staff. However, the majority of these data were retrieved on the following day, maintaining consistency with a prospective data collection approach. Moreover, the proportion of data obtained from the EHR was less than 10%, minimizing its potential impact on the overall findings.
e **Exclusion of the most severe and mildest cases:** The exclusion of patients from ICU, CCU, NICU, and those discharged within 24 hours may introduce selection bias by omitting both the most severe and the mildest cases. This could limit the generalizability of the findings to the broader hospital population.

Despite these limitations, this prospective study offers valuable insights into antibiotic use and consumption in a tertiary hospital in Sana’a, Yemen, contributing to local and regional efforts to optimize antibiotic use to combat AMR.

## Conclusion

This study investigated antibiotic consumption and use patterns in a tertiary hospital in Sana’a. Key findings include a significantly high prevalence of antibiotic use and significant discrepancies between PDD and DDD. The most commonly prescribed antibiotics were Ceftriaxone, Metronidazole, Vancomycin, Levofloxacin, Imipenem/Cilastatin, and Moxifloxacin. To address these challenges, comprehensive antibiotic stewardship programs should be implemented. These programs should focus on regular training and education for healthcare professionals, standardized protocols for empirical therapy, and enhanced surveillance systems. Additionally, promoting rational prescribing practices and improving rapid diagnostic services are crucial for optimizing antibiotic use and combating antimicrobial resistance.

## Supporting information

S1 File Supplementary SPSS dataset in.sav format. (SAV)
